# Safety and efficacy of topical interferon alpha 2B and mitomycin C for localized conjunctival intraepithelial neoplasia: long-term report of their pharmacological safety and efficacy

**DOI:** 10.1186/s12886-023-03092-z

**Published:** 2023-07-27

**Authors:** Beatriz Alvarado-Castillo, Francisco J. Santa Cruz-Pavlovich, Celia Gonzalez-Castillo, Isaac Alejandro Vidal-Paredes, Leonel Garcia-Benavides, Maria Elena Rosales-Gradilla, Jose Navarro-Partida

**Affiliations:** 1 Instituto Mexicano del Seguro Social, Centro Medico Nacional de Occidente, Guadalajara, Jalisco México; 2 Tecnologico de Monterrey, Escuela de Medicina y Ciencias de la Salud, Ave. Eugenio Garza Sada 2501, Monterrey, N.L. 64849 México; 3 Universidad de Guadalajara, Centro Universitario de Ciencias de la Salud, Guadalajara, Jalisco México

**Keywords:** Efficacy, Eye drops, Interferon alpha 2b, Mitomycin C, Ocular surface squamous neoplasia, Safety

## Abstract

**Purpose:**

Ocular surface squamous neoplasia (OSSN) comprises a wide spectrum of squamous tumors, from which corneal/conjunctival intraepithelial neoplasia (CIN) is the most common one. The classic treatment is complete excision, but recurrence rates are high. Antineoplastic drugs such as mitomycin C (MMC) and interferon alpha 2b (IFNα2b) have been used as adjuvants or as primary treatment. To evaluate the efficacy and safety of topical IFNα2b and MMC in patients with CIN, a phase IIb double-blind clinical trial was performed.

**Methods:**

Patients diagnosed with localized CIN were evaluated by slit lamp and impression cytology and were randomly given MMC 0.04% or INF2b (1 million IU/mL) 4 times daily until neoplasia resolution. Time of resolution and frequency of adverse effects were analyzed to determine the pharmacological efficacy and safety of both medications.

**Results:**

Seventeen patients were included. Nine patients were treated with MMC and 8 with IFNα2b. All patients responded to treatment. The resolution time in days was 59.11 ± 24.02 in patients treated with MMC and 143.50 ± 47.181 in those treated with IFNα2b (p < 0.001). In the MMC group, one recurrence was reported (11%). There were no recurrences at 2 years of follow-up in the IFNα2b group. Regarding adverse effects, one or more mild adverse reaction occurred in 77% of patients managed with MMC and in 50% of patients managed with IFNα2b (p > 0.05). No serious adverse effects were reported.

**Conclusions:**

Topical chemotherapy with MMC and IFNα2b demonstrate pharmacological safety and efficacy. Therefore, these drugs could be considered as primary therapies for localized CIN .

## Introduction

Ocular surface squamous neoplasia (OSSN) is a clinical term that denotes a range of conjunctival and corneal squamous epithelial tumors, including conjunctival intraepithelial neoplasia (CIN) and invasive squamous cell carcinoma (SCC) [1, 2]. OSSN is the third most common ocular tumor after melanoma and lymphoma [3, 4]. It is considered a pre-invasive disease and has a prevalence of 0.2 to 3.5 cases per 1,000,000 [4, 5].By definition, CIN is a non-invasive dysplasia confined to the ocular surface epithelium where the basement membrane remains intact and the underlying substantia propria is conserved. CIN can be classified into four categories depending on the degree of epithelial involvement. CIN I refers to mild disease, where the dysplasia is confined to the lower third of the epithelium; CIN II is moderate, with two thirds affected; CIN III has almost the full epithelial thickness involved, and it is carcinoma in situ when the full epithelium is affected [6, 7]. When the atypical cells extend beyond the epithelial basement membrane, the lesion is denominated as invasive SCC [8]. On the other hand, the American Joint Committee on Cancer (AJCC, eighth edition) made a classification system for staging conjunctival squamous neoplasms, which combines the degree of depth invasion and the size and extent of the tumor to surrounding structures [6]. Categories range from T0 (no primary tumor) to T4, which is further divided into T4a, T4b, T4c, and T4d. Using this classification, CIN lesions would get into the Carcinoma in situ category (Tis) (6).

The classical treatment for CIN lesions is surgical excision, followed by cryotherapy. Despite of the immediate and apparent success of the surgical treatment, CIN reemerges with a rate between 9 and 52% [9]. With the purpose of reducing the recurrence rate, different adjuvant therapies have been used including radiotherapy, cryotherapy, chemical agents, and photorefractive keratectomy [10]. Among the chemical agents employed to treat CIN, several antineoplastic drugs can be identified; some of them applied as a first line (primary) treatment, and others as adjuvants, such as, 5-fluorouracil (5-FU), mitomycin C (MMC) and interferon alpha 2b (IFNα2b) [8].

Studies comparing the efficacy of different topical chemotherapy agents as the primary treatment of CIN are limited. With this in mind, the objective of the present study was to compare the long-term efficacy and safety of topical IFNα2b and MMC as primary therapies for the treatment of CIN.

## Subjects and methods

### Patients and study design

In order to evaluate and compare the long-term efficacy and safety of topical IFNα2b and MMC for CIN therapy, a phase IIb, randomized, double-blind clinical trial with a two-year follow-up was conducted. Institutional Review Board (IRB)/Ethics Committee approval from the Instituto Mexicano del Seguro Social (IMSS), Centro Medico Nacional de Occidente, was obtained before enrollment of patients (IMSS Register Number R-2012-785-094). Written informed consent was obtained from all subjects before enrollment. All procedures related to the implementation of this study were supported in the tenets of the Declaration of Helsinki. Sample size was calculated using the Sample Size for Randomized Controlled Trials formula for which an alpha value of 0.05 and a power of 80% were chosen [11]. The standard deviation (σ) was obtained from a previous study [12] and the expected size of effect (d) was 1.73 months. A result of 7 subjects per group was obtained. Written informed consent was obtained from all patients before their enrollment. Additionally, the study was registered in ClinicalTrials.gov, Identifier: NCT02199327. The study included subjects with clinical diagnosis of localized primary intraepithelial neoplasia of the cornea and conjunctiva of any age and gender, with or without systemic comorbidities. Lesions were clinically evaluated and had to be localized with well-defined borders. Patients with lesions that had reached the conjunctival sac, with invasion of the corneal stroma or deepest layers of the conjunctiva, and diagnosis of epidermoid carcinoma with slit lamp and impression cytology were excluded as the treatment of choice in these patients is surgical excision [13]. Subjects who met inclusion criteria were randomly assigned to one of two treatment groups: (1) MMC or (2) IFNα2b group. Eligible patients were blinded about the drug that was provided. The study was performed at the Ophthalmology Department of Centro Medico Nacional de Occidente (Instituto Mexicano del Seguro Social).

### Antineoplastic therapy

Enrolled patients in the MMC group were provided with one dropper (dropper “A”) containing MMC at 0.4 mg/ml (0.04% solution) (Mitolem, Teva, Mexico) and other dropper (dropper “B”) containing saline water at 0.9%. Patients were instructed to apply one drop from the dropper A four times a day by 7 days and one drop from the dropper B by the subsequent 7 days. This cycle of drug and saline drops continued until a full resolution of the lesion was reached. Enrolled patients in the IFNα2b group were provided with two droppers (dropper “a” and “b”) containing solution IFNα2b at 1 million IU/mL (Injection Urifrón, Probiomed, Mexico). Patients in this group were instructed to instill one drop of dropper “a” for the first 7 days and one drop from the dropper “b” the following 7 days. Guidelines were given to all subjects in order to avoid contamination and preserve the cold chain (4ºC). Treatment with IFNα2b solution continued until a full resolution of the lesion was achieved. Treatment failure was defined as lack of response after 3 to 4 cycles of treatment with MMC or failure to achieve complete clinical resolution after up to 6 months of treatment with IFNα2b. Patients who failed to respond to any topical antineoplastic treatment underwent surgical excision.

### Clinical evaluation

Clinical evaluation was scheduled as follows: every two weeks until lesion resolution, and every 4 weeks by 2 years. The ophthalmological examination in each visit included best corrected visual acuity (BCVA), slit lamp evaluation of the anterior and posterior segments, rose bengal and fluorescein stains of the ocular surface and intraocular pressure measurement by applanation tonometry. Additionally, clinical photographs and impression cytology were performed. Clinical photos were used to calculate the size of the lesion and the percentage of tumor reduction using the ImageJ software (Image Processing and Analysis in Java, NIH USA). The extent of the lesions and the clinical examination was accomplished by a licensed ophthalmologist that did not know which of the possible treatments the participant is receiving.

### Impression cytology

Impression cytology is a reliable technique in the detection of OSSN and was performed as previously reported [14]. A Biopore membrane disc (Millipore PICM012550) attached to a small plastic tube was used. The device with the membrane was firmly pressed against the conjunctiva until it became translucent, which takes 10–20 s. The device was then transferred to a container with 96% alcohol. Then the sample cells were sent to the pathology lab, where they were processed and stained with Harris Hematoxylin, and then mounted on a slide. The pathologist did not know the treatment group of each sample.

### Statistical analysis

Analyst was blinded about the treatment group. Quantitative variables were described using mean and standard deviation. Qualitative variables were described using frequencies and percentages. For the contrast of quantitative variables with normal distribution, Student´s t-distribution and paired sampled t-test was used. For the contrast of frequencies, the Chi squared test with Yates correction was used. For the contrast of quantitative variables between groups Mann-Whitney U tests were performed. For the contrast of quantitative variables within groups, the Wilcoxon signed-rank test was used. For comparing repeated measures within groups, Friedman test was done. Kaplan–Meier survival analysis was performed to estimate the survival function of CIN in the MMC and IFNα2b groups. Survival curves comparison was performed by the Mantel-Cox test. A statistically significant *p* value was defined as *p* < 0.05. The statistical analyses were done using the SPSS 22.0 software (SPSS, Inc., Chicago, IL, USA) and graphs were made with GraphPad Prism 9.0.0 (San Diego, California USA).

## Results

Assuming a dropout of 20%, 9 subjects per group were included, enrolling a total of 18 patients with clinical diagnosis of CIN. Nonetheless, one subject from the IFNα2b group was excluded from the analyses as they were later histopathologically diagnosed with invasive SCC. From the remaining subjects, 7 were male, and 10 were female, with a range of age of 50–90 years, and an average age of 70 years ± 11.91. MMC was administered to 9 patients, and IFNα2b to 8 patients. Basal impression cytology was positive in 15 patients (83.3%). Regarding the degree of dysplasia, 7 were reported as mild (16.7%), 7 as moderate dysplasia (38.9%) and 1 as severe (5.6%). All patients were staged as with carcinoma in situ (Tis) using the AJCC classification system. Demographic and clinical characteristics of the included patients in MMC and IFNα2b groups are presented in Table 1. No statistically significant differences were found between groups.

**Table 1:** Summary of demographic and clinic characteristics from the included patients with intraepithelial neoplasms of the conjunctiva and cornea

	MMC	IFNα2B	*p*
Gender			1.0**
M	4	3	
F	5	5	
Age (years)	71.33 ± 14.40	68.75 ± 9.11	0.670 *
Eye			0.347**
Right	6	3	
Left	3	5	
Area (mm^2^)	33.33 ± 24.29	35.17 ± 39.51	0.908 *
Affected meridians	3.22 ± 1.39	4.13 ± 3.27	0.461 *
Disease			0.729**
None	5	6	
HIV	1	1	
Cancer elsewhere	1	0	
Others †	2	1	
Positive initial cytology	6	8	0.206**

HIV, Human Immunodeficiency Virus; IFNα2B, Interferon alpha 2B; MMC, mitomycin C; †, including diabetes mellitus and arterial hypertension; *, Student´s t; **, Fisher exact test.

**Fig. 1:**
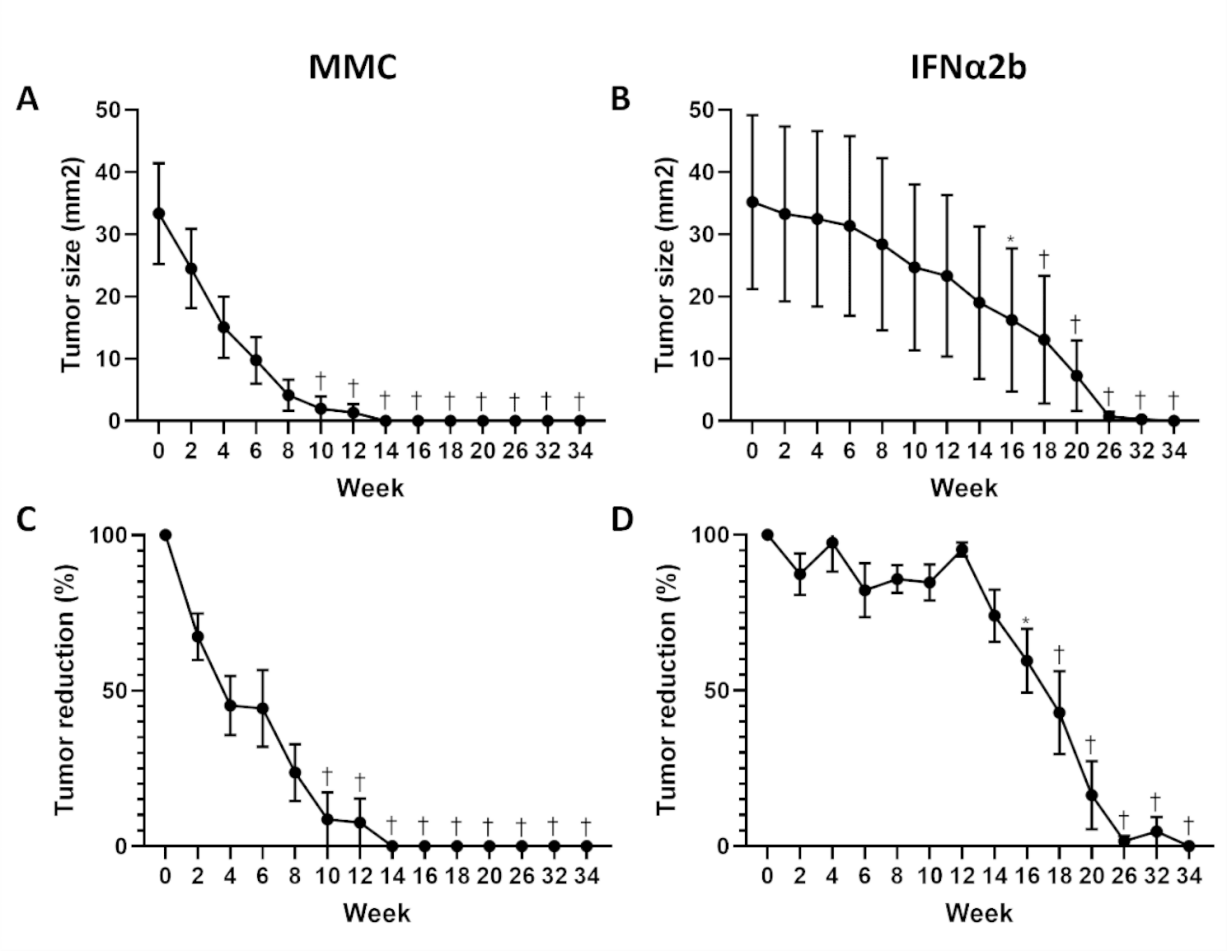
Effects of topical chemotherapy in the size of conjunctival intraepithelial neoplasia. Mean tumor size and percentage of tumor reduction are presented. A consistent reduction in the size of tumor was observed in the MMC group throughout time (**A** and **C**). Similar behavior was observed in the IFNα2b group (**B** and **D**). The shown significant differences are between the respective week and baseline (week 0). The changes in tumor size occurred earlier in the MMC group. Standard error of mean (SEM) is shown. MMC; mitomycin C, IFNα2b; interferon alpha-2b. *, p < 0.05; †, p < 0.01

All 8 patients of the IFNα2b group presented complete resolution of the tumor and no relapses were recorded during the follow-up. Similarly, the subjects receiving MMC also showed complete response to topical therapy; however, one patient presented a relapse 8 months after start of treatment and went through surgical excision. A consistent reduction in the size of tumor was observed in the MMC group throughout time (p < 0.0001). The first significant differences in tumor sizes occurred between week 0 (33.33 ± 24.29 mm^2^) and week 10 (1.97 ± 5.91 mm^2^) (p = 0.004) and continued onwards as shown in Fig. 1A. The percentages of tumor reduction were also significant and can be observed in Fig. 1C. A similar behavior was observed in the IFNα2b group, although in a later time. A significant reduction of tumor size for this group was also shown throughout the study (p < 0.0001) and the first significant difference with week 0 (35.17 ± 39.51 mm^2^) occurred until week 16 (16.22 ± 32.49) (p = 0.02), and also continued onwards until the end of the study. The reductions in tumor size and percentages in the IFNα2b group can be found in Fig. 1B and D.

**Fig. 2:**
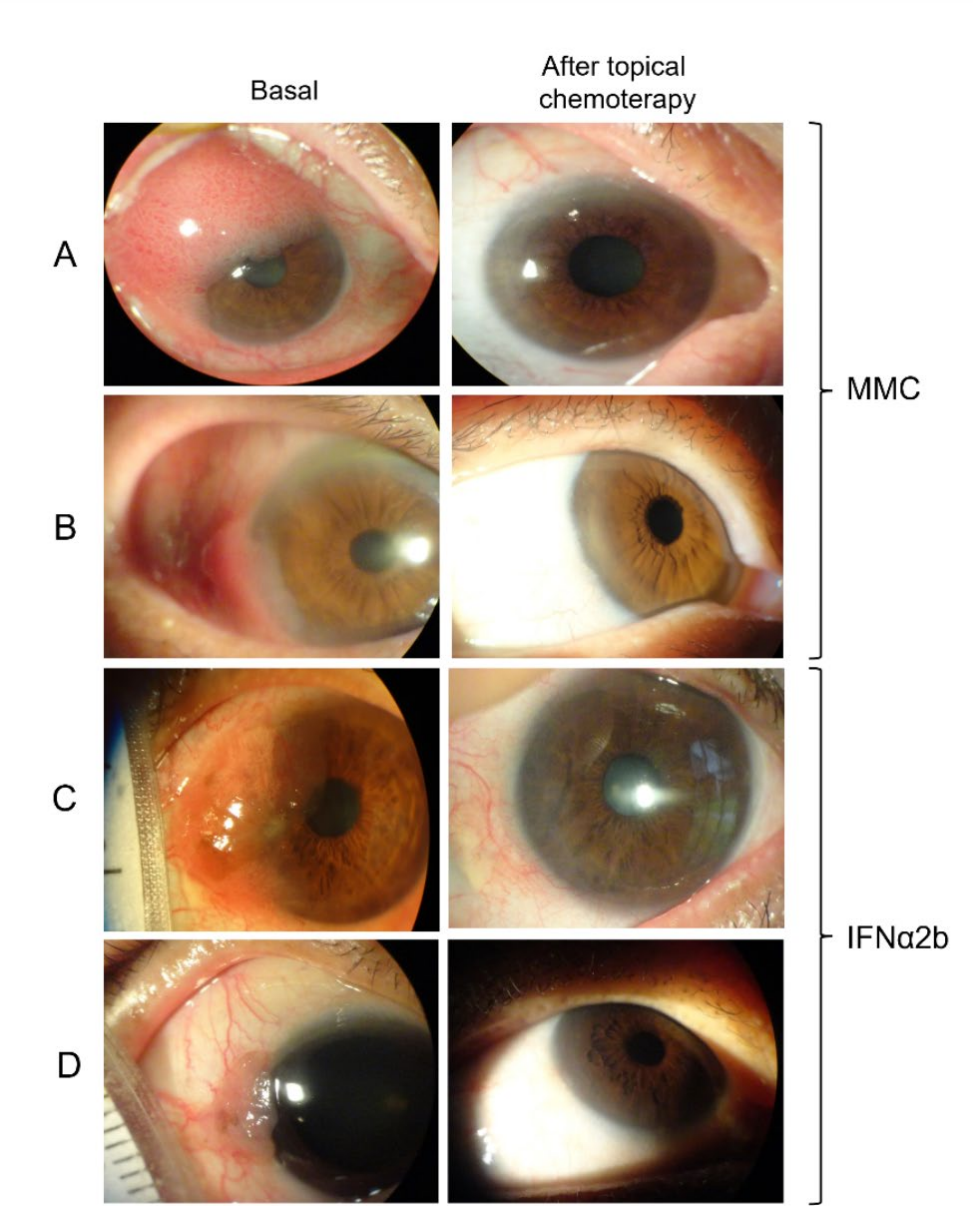
Results of topical chemotherapy for conjunctival intraepithelial neoplasia. Representative cases of topical chemotherapy with MMC (**A** and **B**), and IFNα2b (**C** and **D**) are presented. Patient A required 6 cycles of MMC therapy (84 days) to resolution whereas patient B required 4 cycles (56 days). Patient C required 98 days of IFNα2b therapy to resolution while patient D required 126 days. MMC; mitomycin C, IFNα2b; interferon alpha-2b

The longest time of resolution for IFNα2b was 34 weeks, whereas for MMC was 14 weeks. The mean time for total resolution in the MMC group was 59.11 ± 24.02 days, while for IFNα2b it was 143.50 ± 47.181 (*p* < 0.001). Outstandingly, impression cytology was reported as negative in all patients who presented complete resolution. Representative photographs of the clinical results with topical chemotherapy can be seen in Fig. 2.

**Fig. 3:**
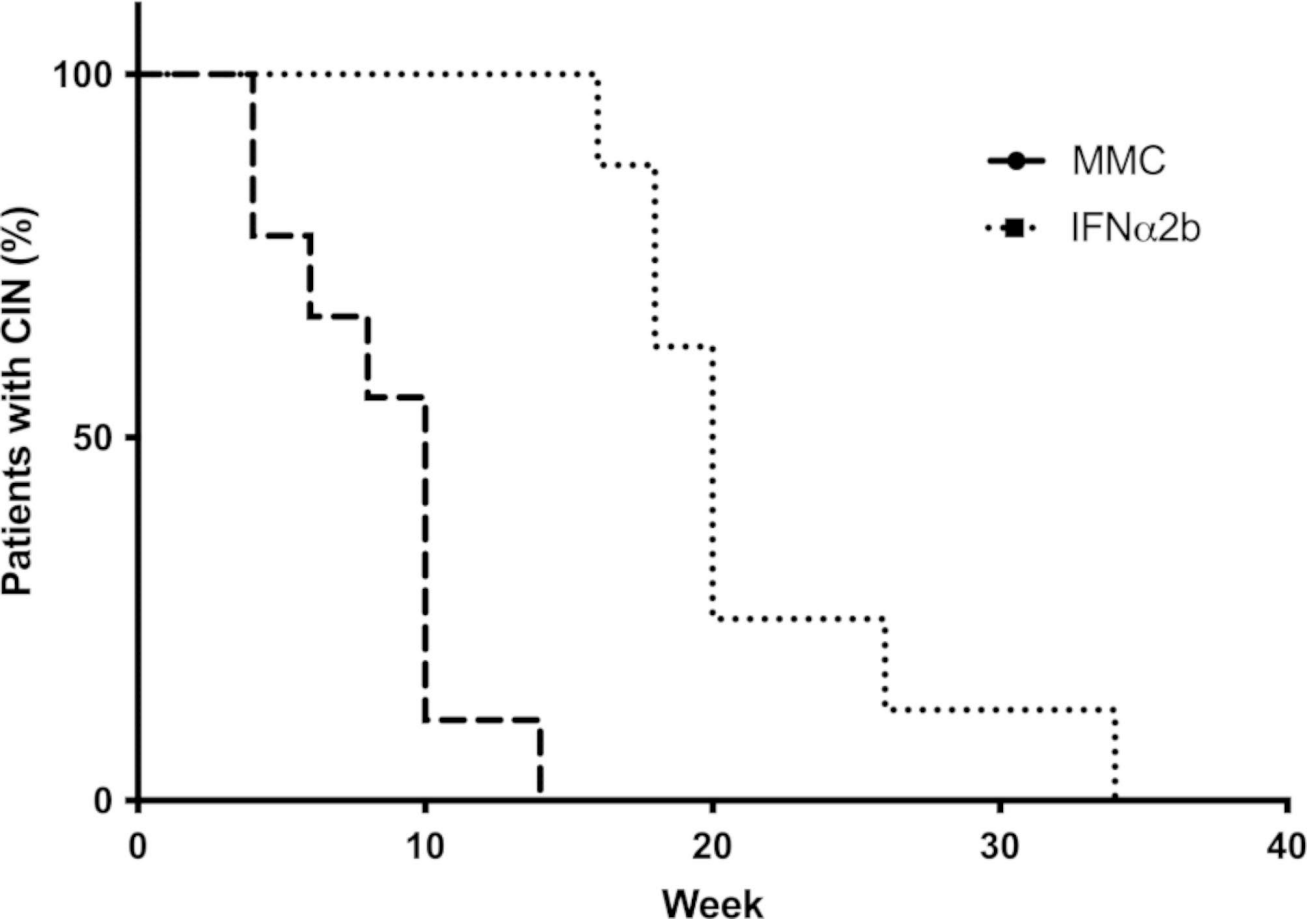
Survival analysis of conjunctival intraepithelial neoplasia treated with topical chemotherapy. The median survival was 10 weeks for MMC and 20 weeks for IFNα2b groups. Survival curves are statistically different (P < 0.0001). MMC; mitomycin C, IFNα2b; interferon alpha-2b

On the other hand, survival analysis of CIN treated with topical chemotherapies revealed that the median survival was 10 weeks for MMC and 20 weeks for IFNα2b group. Survival curves were statistically different (P < 0.0001). The Hazard Ratio (log-rank) was 4.182 (IC 95% 1.307 to 13.38; p < 0.0001) and showed that the tumors treated with MMC have a higher chance to resolve earlier than the treated with IFNα2B. Kaplan-Meier survival plot is presented in Fig. 3.

Lastly, the pharmacological safety of both drugs was similar. Severe side effects, such as corneal ulcer or corneal opacity, were not recorded in any patient; however, the number of reported side effects was greater in the MMC group than in the IFNα2b (16 vs. 5). There were 4 major side effects in IFNα2b and 3 major side effects in MMC. Punctal stenosis and allergy were reported only in the MMC group, whereas follicular conjunctivitis and corneal epithelial microcysts were only observed with IFNα2b topical therapy. Nonetheless, the number of side effects per patient was non-statistically different between groups (*p* = 0.58). The summary of reported side effects is presented in Table 2.

**Table 2:** Reported side effects

Side effects	MMC	IFNα2B	*p*
Foreign body sensation	6 (66%)	1 (12.5%)	0.05
Pain	0	0	NA
Photophobia	0	0	NA
De-epithelization	6 (66%)	1 (12.5%)	0.05
Punctal stenosis	1 (11%)	0	1
Intraocular inflammation	0	0	NA
Allergy	3 (33%)	0	0.206
Follicular conjunctivitis	0	2 (25%)	0.206
Corneal epithelial microcysts	0	1 (12%)	0.471
Total reported side effects	16	5	NA
Number of side effects per patient			0.58
0	2	4	8
1	1	3	
2	3	1	
3	3	0	

IFNα2B, Interferon alpha 2B; MMC, mitomycin C; NA, Not Applicable

## Discussion

The efficiency of antineoplastic drugs as primary therapy in CIN has been documented by different authors. For instance, Parrozzani R. et al. evaluated the efficacy of topical chemotherapy with 5-FU 1% (10 mg/mL, four times/day for 4 weeks) in 41 patients with CIN. Although no adverse events were reported, the rate of recurrence was 7.3% [15]. Meanwhile, Sanket U. et al., reported that the use of IFNα2b (1 million IU/ml, 4 times/day for 6 months) as first line therapy for CIN, was related with complete tumor resolution in 19 of the 23 patients included. However, several adverse effects such as conjunctival hyperemia, follicular hypertrophy, giant papillary conjunctivitis, irritation, corneal epithelial defects, and flulike symptoms were recorded, but resolved within 1 month of medication discontinuation [16]. Moreover, primary topical MMC for CIN has been broadly analyzed. Depending on the study, the dose of MMC varies between 0.002% and 0.04% for desired responses, at the cost of some adverse side-effects [17–21]. Among the most serious ones, limbal cell deficit takes an important spot, being present in up to 12% of patients [22]. A characteristic example of topical MMC for the primary treatment of CIN is the study performed by Ballalai P.L. et al., where MMC 0.02% (0.2 mg/mL, 4 times/day for 4 weeks) was applied to 23 patients. In this study, the rate of recurrence was 4.3% after 24 months of treatment and the principal side-effect was corneal erosion, which developed in 17.4% of patients [21].

To the best of our knowledge, studies about comparative evaluation of available topical chemotherapeutic agents for CIN are limited. In a comprehensive retrospective study, Kusumesh et al. reported the efficacy and safety of MMC versus IFNα2b for the therapy of CIN in 51 eyes of 50 patients. Subjects were treated with either topical IFNα2b (1 million IU/mL, 4 times/day) or MMC 0.04% (0.4 mg/mL, 4 times/ day). In this report, complete response was achieved in 89% of the cases with topical IFNα2b (n = 26) and 92% with MMC (n = 25). The median time to lesion resolution was 3.5 months in the IFNα2b group and 1.5 months in the MMC group, while adverse effects occurred in 12% of patients of the former and 88% of the latter [23].

The need to reduce the risks associated with the surgical treatment of intraepithelial neoplasms has led to the increasingly widespread use of topical chemotherapeutical agents. The direct administration of a drug on the eye avoids the hematoocular barrier, maximizing the drugs’ concentration and limiting its systemic absorption [24]. Most cancer treatments used for ocular tumors are cytotoxic and nonspecific, thus, cancer cells are not directly targeted, causing toxicity in an otherwise healthy eye [24].

According to the present study’s results, MMC has the highest rates of adverse effects, with 77.77% of patients reporting at least one., For the IFNα2B group, rates were lower with 50% of the patients,, however, no statistically significant differences were found between both groups.

MMC has been used as primary therapy in preference to surgical excision, or as adjuvant therapy in surgical removal either preoperative, intraoperative or postoperative with a 82–100% response rate [21, 24–26]. In this study, 100% of the patients responded to the treatment with this antimetabolite.

Regarding the main reported adverse effects in other studies, conjunctival hyperemia (which may be due to cellular toxicity or allergic reaction), pain, burning sensation, superficial pointy keratitis, epiphora, corneal erosion and corneal opacity have been the most reported, being limbic cell deficiency the most severe one [18, 19, 21, 22, 27].

In the MMC group from the present study, foreign body sensation and dotted de-epithelialization were the most reported adverse effects (66%), this in contrast with other studies where de-epithelialization was reported in 17.4% of patients [21]. This may be caused by the posology and dosing, and also due to the recording of very slight changes at the corneal level. In other studies using MMC at a 0.04% concentration (same as in this study), the presence of red eye and irritation was reported in most patients, with the difference that the treatment was administered continuously for 3 weeks instead than in cycles, which may have contributed to such manifestations [28]. In this study’s patients there were no serious adverse effects such as limbic cell deficiency or scleral thinning during the 2 years that they were followed.

On the other hand, topical chemotherapy with immunomodulating agents has gained great attention. Topical IFNα2b has shown to be effective as a single-agent or as an adjunct therapy agent after surgery and since it has not been associated with limbic stem cell damage, it could be an excellent alternative treatment [29]. Secondary effects when used topically are local and minor such as mild conjunctival hyperemia and follicular conjunctivitis [30]. The adverse effects found in the patients of the present study with the IFNα2B treatment were follicular conjunctivitis in 25% of the cases. This manifestation was asymptomatic and was resolved, as in other reports, when the drug was suspended [31]. Among the less common adverse effects, one patient presented microcysts in the cornea, an alteration that has been previously reported, but resolved after the treatment was suspended [32].

When comparing adverse effects individually between those presented with MMC and IFNα2b, there was only a significant difference in the foreign body sensation and dotted de-epithelization, which was expected since most of the reported adverse effects are different according to each drug.

The possibility of administering the MMC continuously or by cycles gives a difference in the time of resolution of the injury. Some studies report a complete resolution within 28 days of continuous administration [21]. In the present study, it was decided to administer the drug in cycles as it has been proposed that treating for one or two weeks, followed by a week of rest, improves tolerance and adherence [30]. Supporting this idea, no patients discontinued the use of the drug; An average of 4.22 cycles of treatment were needed to observe resolution of the lesions, which translates to approximately 59 days in these cycles and 29.5 days of actual administration of the drug, which is similar to the period of time in other studies were treatment was applied continuously. The similitude in the number of days of application could be explained by the fact that cell toxicity is dose dependent [33, 34]. With this in mind, 100% of the patients included in this study presented a complete resolution of the injury. Within this group of patients, one of them (11%) presented recurrence of the lesion after 8 months of treatment, which agrees with other series where recurrence rates from 0 to 10% are reported [20–22, 35]. The recurrences could apparently be related to diffuse injuries rather than localized injuries; however, this is not the only factor that has an influence since in case the patient who presented a recurrence had a localized injury [19].

Regarding the response time, there was a statistically significant difference between both groups, with a longer time in patients treated with IFNα2B. In this group, the average response time was 4.7 months; similarly, other studies report a response from 1 month to 9 months of treatment with an average of 3.25 to 3.5 months [23, 36] [23, 36]. The response rate achieved was 100%, with the same as the reported response rates in similar studies, which go between 80 and 100% and relate with the size and invasion of the lesion [37–39]. In the patients of this study, there was no recurrence of the lesion in the two years of follow-up. The recurrences reported in other series range from 0 to 3.7% and according to the literature there are no statistically significant differences between those treated with IFNα2b and those treated with surgery [23, 38, 40].

It is important to emphasize that the use of topical chemotherapy can be a curative treatment in the management of intraepithelial neoplasms but not for other spectra of ocular surface squamous cell neoplasms, such as carcinoma in situ and invasive epidermoid carcinoma, in which the response to topical treatment is limited [1, 41]. This was the case of one patient from this study, who was initially diagnosed with CIN using the clinical and cytology impression studies and was then excluded from the analyses, as the diagnosis of squamous cell carcinoma was later made due to resistance to treatment and subsequent surgical excision and histopathologic studies.

It should be considered that when there is no response to topical chemotherapy, the lesion being faced could already be considered malignant, in which case resection of the same would be indicated [41]. In the same way, it must be taken into account that, although chemotherapy is not considered to be curative in cases of squamous cell carcinoma, its use is feasible either prior to surgery to reduce the size of the lesion, during, or after the procedure to avoid or diminish recurrences or when the excision has been incomplete [41]. In the case of our patient, chemotherapy was used during the surgical procedure, and, at present, the patient is undergoing two and a half years of surveillance and has no recurrence data.

Another option that has been proposed in patients with resistance to topic chemotherapy is to switch to another antineoplastic agent before considering a surgical procedure [42].

In both groups, the decision of management with topical chemotherapy was based on the clinical characteristics of the lesion; however, in all cases, an impression cytology was performed to confirm the diagnosis. The impression cytology was positive in 83% of the patients, similar to what has been previously reported in other studies [43]. Using a biopore membrane for sampling, a correlation of 80% has been found between the diagnostic impression cytology and the histopathological specimens obtained by incisional biopsy [44, 45]. Similarly, during the follow-up, new cytology was performed when evaluating the clinical resolution. In the subsequent evaluation in the patients who responded to the treatment all the cytologies were negative.

Impression cytology is a diagnostic method with high predictability, but in the case of malignant keratinizing neoplasms the possibility of false negatives must be considered due to the scarcity of cells in the sample, so care must be taken in the interpretation in these cases [46].

In 2013, surveys carried out by cornea specialists regarding the standard of choice for the treatment of squamous neoplasms of the ocular surface demonstrated an increase in the use of topical chemotherapeutic agents [1, 47]. The arrival of new diagnostic methods such as high-resolution optical coherence tomography (*HR-OCT*) has brought the opportunity to guide the management in a more objective manner, as it can identify residual subclinical disease in cases with apparent clinical resolution [48, 49]. The use of these technologies has been rising a tendency for the use of topical chemotherapeutic agents as the primary treatment for squamous neoplasms of the ocular surface [1, 8].

For future studies, consideration should be given to conducting a study with a larger number of patients and having a longer-term follow-up in order to assess the possibility of adverse effects that do not occur in this follow-up time and also to determine the presence of later recurrences.

## Conclusions

Topical chemotherapy with MMC or IFNα2b could be a safe alternative to surgery with good efficacy and safety, with low recurrence rates. Both chemotherapeutic agents offer a similar efficacy, the duration of treatment being the only difference between the two. Similar studies comparing different chemotherapy modalities, with a higher number of patients and ideally a longer follow up should be done.

## Data Availability

The datasets used and/or analyzed during the current study are available from the corresponding author on reasonable request.
